# GP’s role in supporting women with anal incontinence after childbirth injury: a qualitative study

**DOI:** 10.3399/BJGP.2023.0356

**Published:** 2024-06-11

**Authors:** Abi Eccles, Joanne Parsons, Debra Bick, Michael RB Keighley, Anna Clements, Julie Cornish, Sarah Embleton, Abigail McNiven, Kate Seers, Sarah Christine Hillman

**Affiliations:** Warwick Applied Health, Warwick Medical School, University of Warwick, Warwick.; Warwick Applied Health, Warwick Medical School, University of Warwick, Warwick.; Warwick Clinical Trials Unit, University of Warwick, Warwick.; Nottingham.; Nottingham.; Department of Colorectal Surgery, Cardiff and Vale University Health Board, Cardiff.; Nottingham.; Nuffield Department of Primary Care Health Sciences, University of Oxford, Oxford.; Warwick Applied Health, Warwick Medical School, University of Warwick, Warwick.; Warwick Applied Health, Warwick Medical School, University of Warwick, Warwick.

**Keywords:** general practice, incontinence, primary health care, women’s health

## Abstract

**Background:**

Obstetric anal sphincter injury is the most common cause of anal incontinence for women, which often has profound impacts on women’s lives. GPs offer a first line of contact for many women, but we know that very few women experiencing anal incontinence postnatally report discussing it with their GPs.

**Aim:**

To identify key ways in which GPs can support women with anal incontinence caused by childbirth injuries.

**Design and setting:**

A qualitative study investigating women’s experiences with their GP, and GPs’ perspectives about providing such care.

**Method:**

This qualitative study combined two phases: first, a series of in-depth semi-structured interviews with women experiencing anal incontinence caused by childbirth injuries (*n* = 41); and second, focus groups with GPs (*n* = 13) stratified by experience. Thematic analysis was conducted and relevant themes from across the two datasets were examined.

**Results:**

Mediating factors in GP care for women with anal incontinence caused by childbirth injuries centred around three key themes: the role of the GP, access and pathways, and communication.

**Conclusion:**

The findings demonstrate multifactorial challenges in identifying the problem and supporting women experiencing anal incontinence after childbirth injury in primary care settings. Many GPs lacked confidence in their role in supporting women, and women were often reluctant to seek help. Those women who did seek help often experienced frustrations consulting with their GPs. In a context where women are often reluctant to ask for help, their concerns are not always taken seriously, and where GPs do not routinely ask about anal incontinence, potential anal incontinence after childbirth injury appears to be often missed in a primary care setting.

## Introduction

Anal incontinence is the inability to control bowels and/or flatus,^1^ is associated with faecal urgency, and is estimated to affect 1–10% of adults.^2^ It is more prevalent in women, with 20% experiencing anal incontinence,^3^ most commonly caused by obstetric anal sphincter injury.^4^ Anal incontinence resulting from childbirth injury often has profound impacts on women’s day-to-day lives, identity, and emotional wellbeing.^5^^,^^6^ Many women experience anal incontinence soon after giving birth, but some have worsening or even new-onset symptoms at the time of the menopause.^7^ However, despite such prevalence, there are no clear follow-up pathways for women experiencing anal incontinence when the injury is not identified immediately during or after childbirth.^8^

For most women, their GPs will be first line for suggesting a likely diagnosis, as well as support and management, of anal incontinence. This wider support could include advice related to diet and self-management, and signposting to resources. Women may benefit from onward referral to specialists such as women’s health physiotherapists, gynaecologists, and colorectal surgeons. However, we know that very few women experiencing anal incontinence postnatally report having discussed this with healthcare professionals, and that many women are not asked by their GPs about anal incontinence during routine postnatal checks.^9^ In 2022, the *Women’s Health Strategy for England*^10^ highlighted that postnatal care is lacking, and that women’s concerns are often dismissed, and their pain normalised, by healthcare professionals. Such a context suggests that there are likely to be many women with anal incontinence after childbirth injury who are not accessing care and support. This study examines this gap by exploring the experiences of women in relation to contacting their GP, and GPs’ perspectives about providing care to such women. This article refers to women; however, the concepts herein apply to all birthing people.

**Table:** How this fits in

Anal incontinence after childbirth injury has profound impacts on women’s lives and many women find they cannot access health care and support. GPs can play a crucial role, but it is known that very few women speak to GPs about their symptoms. In combining GPs’ perspectives and the views of women affected by anal incontinence, this study shows how anal incontinence after childbirth injury is often missed in a primary care setting. Drawing on these findings, some key ways are highlighted in which GPs can provide support for such women.

## Method

This qualitative study combined two phases. First, a series of in-depth interviews were conducted between October 2021 and May 2022 with 41 women who had anal incontinence resulting from childbirth injury. Second, focus groups with GPs were conducted by video call in March 2022 (see Supplementary Box S1 for details). Using a narrative approach, we explored those women’s experiences and access to healthcare services, and GPs’ experiences of, and views about, providing care to women with such injuries. Throughout the study we worked closely with two women who are injured mothers and work closely with the MASIC Foundation, a charity that supports women who have sustained birth injuries. As co-investigators on the study, the two women attended regular study meetings; provided feedback on study documents; piloted the interview schedule for injured women participants (see Supplementary Box S2 for details); commented on findings; helped develop dissemination materials; advised on terminology; co-authored articles; and generally provided their perspective as and when relevant.

The fieldwork was carried out by two qualitative health researchers with guidance from the study’s principal investigator. Interview participants were recruited via hospitals and social networks of related organisations, and received a shopping voucher as reimbursement for their time. Women were eligible if they were >18 years and had anal incontinence caused by childbirth injury. Women <18 years, those unable to provide consent, and those with no history of a vaginal birth were excluded from the study. Using a maximal variation approach, we purposively sampled 41 participants with aims to ensure a diverse sample in terms of ethnicity, socioeconomic status, and age. We also sampled those experiencing anal incontinence within 7 years of childbirth, as well as those with worsening (or new) symptoms around the time of the menopause. Table 1 gives demographic details of the women participants. Women were interviewed via video call (*n* = 33) or phone (*n* = 8) according to their preference. Thirteen GPs were recruited via social media and networks, and stratified into three focus groups according to their number of years practising as a GP (group A: five with <5 years’ experience; group B: five with ≥5 years’ experience; group C: three trainees) (Table 2).

**Table 1.** Background characteristics of injured women participants

**Characteristic**	**Women experiencing anal incontinence**	
	**Within 7 years of childbirth (*n* = 25)**	**Around time of menopause (*n* = 16)**
**Mean age, years (range)**	36 (20–47)	55 (41–75)

**Ethnicity, *n* (%)**		
White British	17 (68)	14 (88)
White Polish	2 (8)	
White other	2 (8)	
British Asian	3 (12)	
British Pakistani		1 (6)
British Indian		1 (6)
Black Caribbean	1 (4)	

**Deprivation score, *n* (%)**		
1st quintile	6 (24)	2 (13)
2nd quintile	5 (20)	7 (44)
3rd quintile	3 (12)	2 (13)
4th quintile	7 (28)	3 (19)
5th quintile	4 (16)	2 (13)

**Table 2.** GP participants’ characteristics

**Characteristic**	
**Experience, *n***	
<5 years practising as a GP	5
≥5 years practising as a GP	5
Trainee	3

**Gender, *n***	
Female	11
Male	2

**Mean Age, years (range)**	39 (29–44)

**Practice deprivation, *n***	
Affluent	3
Mixed	4
Deprived	6

**Practice rurality, *n***	
Rural	3
Mixed	1
Urban	9

### Data analysis

Interview and focus group recordings were transcribed verbatim, anonymised, and uploaded to NVivo v13. Guided by reflexive thematic analysis,^11^ two researchers carried out familiarisation with the datasets, followed by coding and initial theme generation. The themes were then developed and reviewed in discussion with the wider team, and finally themes were refined and defined. A proportion of data were dual coded to scrutinise quality of code development.

### Combining data

The women we spoke with discussed their experiences in depth during interviews, and the findings about their broader experiences are presented elsewhere.^12^^,^^13^ For this article, interview data organised into codes relevant to primary care were exported and analysed using the One Sheet of Paper (OSOP) method.^14^ The OSOP analytical method allows researchers to map and group content, and identify patterns, within coded data to create a conceptual map. This conceptual map was examined alongside the themes developed from analysis of focus group data, and overarching themes across both datasets were identified and summarised. These were scrutinised and refined with the wider study team.

## Results

The women we spoke with had varied experiences when consulting their GPs. Many described difficulties they encountered, while others referred to times they felt particularly supported, and some talked about not consulting their GP about this issue.^13^ All the GP participants were highly engaged and interested in discussing how to help women, but their confidence levels varied in how to support women with anal incontinence. GPs with >5 years’ experience discussed the complexities of such consultations and the issues entailed, with some referring to previous consultations with women with this presentation.

In-depth analysis of the women’s and GP datasets allowed further exploration of nuances of how GP care is accessed and carried out in reality. We identified themes that were grouped into three overarching areas: the role of the GP, access and pathways, and communication during consultation.

### Role of the GP

We identified three subthemes related to the role of the GP: confidence in the GP role for women with anal incontinence, the constraints of primary care, and the importance of listening and ‘sowing the seed’ so women re-consult.

#### Confidence in the GP role for women with anal incontinence

GP participants varied in confidence when discussing how they would support women with anal incontinence caused by childbirth injury. GPs with >5 years’ experience spoke most confidently, drawing on their experience of previous consultations. Some GPs talked about how they had not encountered this presentation and lacked confidence in the best way to support women. The lack of direct experience for some GPs meant that they spoke about consultations in hypothetical terms and how they would seek advice from colleagues:

*‘I’m pretty much out of my depth to be honest so I think I would get some advice and guidance.’*

(GP, A1)

Some participants felt their GPs supported them well, listened, and referred them appropriately. Others did not feel confident that their GP knew how to support them and were frustrated with previous unsuccessful consultations. In some cases, women saw GPs as gatekeepers to appropriate care, rather than a source of health care and guidance, and many said they independently gathered information and decided on their preferred course of action before seeing their GP:

*‘My experience shows that when I go to the GP, I already need to know where I want to go in a way.’*

(Woman, p.33)

Across all focus groups, GPs concurred that their training in this presentation was lacking or non-existent. Some noted that they felt more confident and well trained in consulting women with urinary incontinence resulting from childbirth injury than with anal incontinence. They highlighted that any relevant training was limited to secondary care, which is not always relevant to how women would present in primary care:

*‘We get taught about, you know, urinary incontinence, stress, versus, you know, urgency and things like that, but there isn’t really anything targeted for faecal incontinence.’*

(GP, A1)

#### Constraints of primary care

There was recognition from both women and GPs that this presentation typically required specialist input. When acknowledging their lack of confidence, some GPs noted long delays for secondary care, meaning that women needed interim treatment and support.

GPs also highlighted lack of primary care resources as a key limitation to providing care. They talked about how other issues may take priority during consultations, giving examples of safeguarding, babies’ needs, and lack of time. Some also talked about how continuity and follow-up care was challenging:

*‘*[There was] *a woman who spoke entirely Pashto. There was no interpreter arranged, so she phoned her daughter five minutes into the consultation. And we then had to conduct it three-way on the telephone. And halfway through this, doing the, her postnatal, I discovered that her baby had Down’s syndrome and had a whacking murmur. And so when she asked me to examine her perineum, I basically had to say no. And I’m, I’m still feeling bad about it*.*’*

(GP, B5)

#### Importance of listening and ‘sowing the seed’ so women re-consult

It is apparent from women’s and GPs’ accounts that anal incontinence caused by childbirth injury typically takes a long time to be recognised by both the women themselves, and the healthcare professionals they consult. The reasons for this include uncertainty as to whether symptoms are ‘normal’ after childbirth and likely to resolve without intervention; prioritisation of the baby’s needs over that of the mother’s; confusion with other diagnoses, such as irritable bowel syndrome (IBS) or potential cancer; and women’s reluctance to disclose embarrassing symptoms.

One key finding was the role that GPs could play in ‘sowing the seed’ to enable women to feel they could re-consult. Some of the more experienced GPs discussed reading tacit cues in women reluctant to disclose anal incontinence, by their body language and phrasing. Efforts to listen and make women feel comfortable were rewarded when women booked a follow-up appointment to address the problem. There were examples in women’s accounts where a GP who took the time to listen helped them to return for help:

*‘*[The patient] *wasn’t very complete in her answer … so I could tell … she was resistant of, to talk much further about it. And I think I said something like, “Well, if you were having problems with your bowels, there may well be things we could do to help you.” And that obviously planted a seed in her mind, because she didn’t talk about her bowels on that consultation, but she did return a couple of weeks later*.*’*

(GP, B4)

*‘Just to listen to people and understand that effects and physically, mentally, emotionally … She didn’t force anything on me. And two weeks later I’m ringing her back*.*’*

(Woman, p.36)

### Access and pathways

Key areas influencing access and pathways were inadequacy of postnatal checks, uncertainty about pathways, lack of follow-up, and proactive/reluctant women.

#### Inadequacy of postnatal checks

GP postnatal checks typically take place around 6–8 weeks after birth. An issue identified in both GP focus groups and women’s interview data was how babies’ needs are prioritised, meaning there is less impetus, and limited time, to address any issues that women might be experiencing. Some of the GPs talked about ensuring there is a dedicated appointment for the woman. However, others judged that in some cases women would be less likely to attend if separate appointments were made for them and their baby.

GPs agreed that limited time during postnatal checks was a barrier to comprehensively addressing issues women may be facing and they are often unable to carry out an internal examination within that time. Many GPs said they did not routinely ask about anal incontinence at postnatal checks unless there was a specific reason (for example, a third-degree tear was recorded in the notes) and that it was not part of the standardised checklist. Many GPs felt the timing of the postnatal check at 6–8 weeks was too early, because of the potential that problems were temporary, women’s focus on their babies’ needs, and a possibility that women are not ready to disclose problems:

*‘If I try to deal with it all in that six to eight week check I’d worry that I’d just be, kind of, rushing through it massively*.*’*

(GP, A3)

*‘That postnatal six-week GP check. It was probably about eighty per cent about the baby really, and maybe twenty per cent about me … I felt like, “Oh, I’ve got another patient and I really, really need to rush you through” and I still didn’t really touch on half of the stuff I was experienci*ng.’

(Woman, p.33)

Notwithstanding the importance of having a routine GP appointment for postnatal women, there was a strong view that the time constraints and complexity of postnatal checks meant the current set-up was not adequate.

#### Uncertainty about pathways

The complexities of women’s circumstances and differences in local services mean that a ‘one-rule-fits-all’ pathway is unavailable. Thus, it was unsurprising that there was uncertainty regarding subsequent onward referral. More experienced GPs drew on previous consultations and explained how women’s circumstances influence referral decisions. For example, surgery may not be suitable for women who want more children, and older women are more likely to be referred on to a 2-week wait pathway to determine if their symptoms are caused by cancer.

There was also a lack of clarity regarding what would happen once referrals were made. Some GPs were concerned about ‘overpromising’ services in secondary care and so limited the information that they gave women. GPs also highlighted that long delays to care meant it was unclear when women would access the services they needed, leaving GPs to help women in the meantime:

*‘Locally, for us there are routine gynae referrals that got to two years where they’re seen at the moment and certainly that would be wholly inappropriate for this kind of issue.’*

(GP, A5)

Some women were unaware of which services would help them and found that their GPs did not appear to know either. Others did not expect their GPs to know, and so did their own research and visited their GP with a referral in mind. Some women, aware of the delays to secondary care, decided to access care privately instead.

#### Lack of follow-up

GPs recognised that the ‘red flag’ pathway meant that, if test results ruled out cancer, many women did not have a subsequent appointment to readdress the anal incontinence. Overstretched resources mean practices are unable to follow up such patients and often GPs are unaware of negative test results processed by administrative teams. This was apparent in some women’s accounts, who found there was no follow-up after testing for cancer. Similarly, when some women felt their GPs judged their symptoms to be ‘normal’ they were reluctant to return. The lack of robust follow-up with women experiencing anal incontinence relies on women to take the initiative to make follow-up appointments to revisit the problems they are experiencing:

*‘*[GP] *referred me quickly because they wanted to rule out cancer … The only thing is, is where I’ve still got the issue*.*’*

(Woman, p.19)

#### Proactive/reluctant women

Access to care was in part influenced by how proactive or reluctant women were in seeking help. Some women talked about how they were proactive when trying to access care, by being assertive, independently gathering information and preparing for consultations, returning when issues were not addressed, and, in some cases, consulting a different GP:

*‘I felt very self-motivated that if I’d not kept complaining it wouldn’t have happened and then I think it was very hard to access*.*’*

(Woman, p.6)

Other women were reluctant to seek help from their GP. Some talked about how they found their symptoms embarrassing and so found it difficult to discuss with their GP. Some were reluctant as they did not think there would be help available, or they had previously been told their symptoms were ‘expected’ or ‘normal’ after childbirth. Some put it off and talked about mentioning it next time they were in for another appointment:

*‘I’ve kind of just put it on a on a on a hold … I’ll probably will mention it when I when I get a face-to-face consultation, but to be to be honest I just live with it. I just put up with it*.*’*

(Woman, p.21)

GPs also recognised that women were often reluctant in coming forward. They talked about the stigma associated with anal incontinence and compared it with urinary incontinence, which is more commonly discussed. GPs recalled consultations where women described the problem as ‘disgusting’ and one woman who ‘had to apologise profusely before she could even talk to me about it’. They also felt that some women do not know help is available and may delay seeking help while trying to cope alone:

*‘I haven’t seen lots of women with anal incontinence, I’ve definitely seen women with other vulval conditions or urinary incontinence who have said, you know, “I’ve never done anything about this because I didn’t think there was anything I could do. I thought this was normal, I just have to live with it. No one’s ever told me anything different.” And I think that probably, would be my guess, would also apply to faecal and flatus incontinence*.*’*

(GP, C1)

### Communication during consultation

GP participants across all three focus groups recognised that the apparent rarity of anal incontinence following a childbirth injury may be because it is going undetected. They acknowledged that the way in which GPs communicate during consultations can be a key factor in enabling women to present with anal incontinence, and for them to identify the problem. Three subthemes were identified in relation to communication: the need for clear, sensitive discussion, long-term listening and taking time, and language barriers.

#### Need for clear, sensitive discussion

As women may be reluctant to come forward, GPs recognised the importance of the GP initiating a conversation about potential anal incontinence with postnatal women. However, they also recognised that there was a balance between clearly noting potential problems while being sensitive, as some women may need careful encouragement. The importance of sensitive, but direct, discussion was reinforced by interview data. Some women talked about instances when their GP had been insensitive, and they found this upsetting, while others talked about how they appreciated it when their GP spoke directly about their issues as they felt they were being taken seriously:

*‘*[My GP] *crossed her legs like this and said, “That gives me the creeps.” … that made me really upset*.*’*

(Woman, p.38)

*‘It’s embarrassing enough anyway without then struggling to find the words … Typically, medical professionals can be quite direct and I, you know, I understand they’re trying to get the facts and everything, but I think that can sometimes then make you withdraw into yourself*.*’*

(Woman, p.8)

Responses to such discussion are likely to vary between women, and balancing of clear yet sensitive language while being responsive may be challenging for GPs.

#### Long-term listening and taking time

Potential for other causes (such as IBS or cancer) and the period of recovery after childbirth means there is often uncertainty initially as to whether symptoms have other causes or will improve without intervention (see ‘Lack of follow-up’ section above). Thus, anal incontinence caused by childbirth injury may, therefore, not be diagnosed during an initial consultation and may require multiple consultations.

However, many women were left feeling that their concerns were not taken seriously, were dismissed as being something else, and that their problems were normalised. This led to some internalising their problems, feeling alone, and not re-consulting. Many older women who had had a test result ruling out cancer did not consider returning to the GP with an issue that they felt had already been investigated.

Some GPs recognised the importance of taking the time to listen, and women talked about times that had been particularly useful for them. In line with the ‘sowing the seed’ subtheme, it appears to be important for GPs to make it clear to women that they are being taken seriously and to encourage women to make a follow-up appointment:

*‘One of the key things is being, being that doctor that listened. And if you were once that doctor that listened, that is probably the only way you’re gonna get that patient back*.*’*

(GP, B5)

*‘I’ve told him* [my GP] *what level of tear I had and, and how traumatic it was. But again, even with that I felt like he took no notice.’*

(Woman, p.33)

#### Language barriers

GPs highlighted times when language barriers between themselves and women whose first language is not English made consultations particularly difficult. It was not always clear if patients had understood advice, and following up with the patient was challenging:

*‘Her consultations with me were often about different things but would always also be about this* [anal incontinence]. *And it was always very difficult to understand what her understanding was of when this problem could be fixed. And she wanted more children, but she also wanted to have the operation before she was having more children. And it was, you know, these, sort of, circular consultations.’*

(GP, B3)

Some women also highlighted a problem with not understanding medical terminology, so hindering their understanding of the problems and advice:

*‘They were saying to me, “Oh well, it’s your internal sphincter.” … I didn’t have a clue what that is. What does that do? What does that mean? And you know, I’ve had to go away and find out for myself.’*

(Woman, p.8)

Many women said that they did not understand information during the consultation and that they spent time later gathering their own information. Discussion in lay terms during consultations may, therefore, help women to understand and give opportunities for further questions.

## Discussion

### Summary

It is clear there are missed opportunities when anal incontinence after childbirth injury could be detected in various healthcare settings.^13^ GP consultations offer an opportunity to initiate care; these findings combine women’s and GPs’ perspectives to provide unique insights into the reasons why it is often not identified by GPs and ways in which care can be improved.

Postnatal women are not routinely asked about anal incontinence, and GPs are unsure if it is appropriate to do so at every postnatal check. GPs often do not feel confident and report a lack of training in this area. Women are often reluctant to seek help, and those who are proactive in seeking help often feel as though their concerns are not taken seriously. Adding to the confusion, anal incontinence may be caused by other issues, such as IBS or cancer. Some women find the cause is misdiagnosed as IBS, which then limits access to appropriate advice and secondary care.^13^ Findings from the interviews and focus groups suggest a lack of robust follow-up for women referred on to a 2-week-wait pathway to rule out cancer, which typically relies on women (who may have been previously reluctant to seek help) to re-present to their GP for further assessment after receiving their negative cancer test result.

During the focus groups, GPs recognised having insufficient time for complex postnatal consultations, a lack of robust follow-up, and women’s reluctance to talk about anal incontinence as contributing to missed diagnoses. Drawing on their own experiences, some GPs discussed the importance of showing they are listening and ‘sowing the seed’ by mentioning potential anal incontinence and that there is help available. In doing this, women appear more willing to return if they are experiencing issues.

There was uncertainty regarding the GP’s role in supporting women identified across both datasets, suggesting a lack of awareness regarding the support GPs can provide. However, this study demonstrates the value of sensitively initiating discussions (‘sowing the seed’) about anal incontinence during the postnatal check (or equivalent) to encourage women to feel more comfortable in disclosing their issues. GPs can also remind women to make follow-up appointments when sending a test for cancer, to ensure follow-up. The Royal College of General Practitioners — in conjunction with the research team — recently released an online learning tool that provides a useful resource for GPs and other healthcare professionals to increase their knowledge and understanding in how they can support women with this condition.^15^

### Strengths and limitations

In combining women’s and GPs’ perspectives, this study provides a balanced and comprehensive overview from multiple perspectives of challenges facing women with anal incontinence after childbirth injury and ways to support them in primary care. Qualitative methods allowed in-depth exploration of their experiences and the complexities of such consultations. One-to-one in-depth interviews enabled a good rapport to be gained between women and the researcher, and women appeared to be open and comfortable when sharing their experiences. This was the case for both modes of interview: video calls and phone calls.

We interviewed women from a range of backgrounds. While we translated posters, advertised via ethnic minorities women’s organisations, community groups, and employed an interpreter to invite women to take part, we did not manage to include any women who were not fluent in English.

As GPs volunteered to give up their time to take part in focus groups, it is possible that participants were particularly engaged GPs with an interest in women’s health. This allowed thoughtful discussion but may mean that other perspectives were missing. Nonetheless, the fact that this group highlighted omissions in knowledge and confidence is suggestive that this may be the case even more so for GPs without a women’s health interest.

### Comparison with existing literature

Women’s feelings of embarrassment and shame are well documented,^5^^,^^16^^,^^17^ and surveys have shown that women rarely seek help from healthcare professionals about the problem of anal incontinence.^18^^,^^19^ Our findings also show how women were reluctant to seek help because of prioritising their babies’ needs and feeling embarrassed to talk about anal incontinence. Conversely, there were also examples of women who were particularly proactive in seeking help, gathering information, making repeated visits to their GP, and deciding their preferred course of action before the consultation. This may be reflective of a sample that included a higher proportion of affluent women, who are more likely to be health literate,^20^ and confident during medical consultations.^21^

Previous studies show that most postnatal women are not asked about anal incontinence by healthcare professionals,^8^^,^^9^ and therefore it was unsurprising that most GPs said they would not routinely ask about anal incontinence and it was often not incorporated in their postnatal checklists. During deliberations, GPs questioned whether it is appropriate to always initiate such discussions during postnatal checks. Current National Institute for Health and Care Excellence (NICE) guidelines for postnatal care recommend that GPs ask about bowel incontinence, but this is among a myriad of other items with little prominence.^22^ Our findings also showed how GPs find there is typically a lot of content to cover during postnatal checks with little time available, and language barriers (not only relating to women who do not speak English but also to those with limited understanding of medical terminology) add complexity to such consultations.

Women often felt like their concerns were not taken seriously and were framed as being a ‘normal’ part of recovery from giving birth. The NICE guidelines state that *‘there does seem to be some spontaneous resolution of symptoms’* for women with anal incontinence after childbirth,^2^ whereas others argue it is not a ‘normal’ consequence of giving birth and women ought to be empowered to seek help.^8^ Inconsistency in information suggests some confusion among professionals. Normalisation is seen more broadly, as the *Women’s Health Strategy for England* recently showed that women often feel that they are not listened to by healthcare professionals and their symptoms are normalised as ‘to be expected’.^10^

This study showed examples of proactive women who sought help, and GPs who took concerns seriously and sensitively asked women about potential anal incontinence. However — based on the overall study findings and literature — this appears to be exceptional rather than routine. In a context where women are often reluctant to ask for help, their concerns are not always taken seriously, and, where GPs do not routinely ask about anal incontinence, it is easy to see how potential anal incontinence after childbirth injury is often missed in a primary care setting.

### Implications for research and practice

GP consultations can provide a first point of contact for access to care and referrals, but these findings demonstrate multifactorial challenges in identifying the problem and supporting women experiencing anal incontinence after childbirth injury in primary care settings. As discussed, there are various issues with postnatal checks and lack of robust follow-up in detecting women with this issue. Ideally, GPs would have more time and resource to address these issues, but this is not the reality of modern primary care where resources are often stretched and there are other issues to contend with (such as other health issues, the baby’s health, language barriers, and a reluctance to discuss). In such a setting, providing adequate care is problematic and, without addressing the broader issue of overstretched resources, it is hard to see how this could be overcome.

As with many areas of healthcare access, diagnosis of anal incontinence caused by childbirth presents a particular challenge in women from certain demographic groups. In the UK, women from less affluent backgrounds, and teenagers, are less likely to attend postnatal checks.^23^^,^^24^ We also know that women with Asian ethnicity are significantly more likely on average to sustain such injuries during childbirth,^25^^,^^26^ thus focus on support and research for specific groups may be beneficial.

Drawing on the study findings, there are key ways that GPs can support women with anal incontinence after childbirth injuries, some of which are also detailed in a Royal College for General Practitioners eLearning module: *Anal Incontinence in Women with Previous Childbirth Injury*.^15^ These include:

- asking women directly about anal incontinence and difficulty controlling bowels after childbirth, and asking on more than one occasion, which may help women to voice their concerns;
- at the point when women are referred to the 2-week-wait pathway to test for cancer, clearly advising them to subsequently re-consult after a negative cancer result;
- being aware that Asian women are at higher risk and acknowledging potential cultural differences in discussing sensitive subjects. An independent translator should be used wherever possible for patients who do not speak English;
- being aware that the biopsychosocial implications of anal incontinence after childbirth can be profound and should be explored;
- not suggesting that anal incontinence is normal, but instead giving simple advice (pelvic floor exercises, diet) and making referrals to a women’s health physiotherapist/pelvic floor clinic, tailored to individual need and severity of symptoms, where possible; and
- signposting women to charities that provide support, such as the MASIC Foundation (https://masic.org.uk/) and the Birth Trauma Association (https://birthtraumaassociation.org/home).

## References

[1] Sultan AH, Monga A, Lee J (2017). An International Urogynecological Association (IUGA)/International Continence Society (ICS) joint report on the terminology for female anorectal dysfunction. Int Urogynecol J.

[2] National Institute for Health and Care Excellence (2007). Faecal incontinence in adults: management CG49.

[3] Sharma A, Yuan L, Marshall RJ (2016). Systematic review of the prevalence of faecal incontinence. Br J Surg.

[4] Jorge JM, Wexner SD (1993). Etiology and management of fecal incontinence. Dis Colon Rectum.

[5] Keighley MRB, Perston Y, Bradshaw E (2016). The social, psychological, emotional morbidity and adjustment techniques for women with anal incontinence following Obstetric Anal Sphincter Injury: use of a word picture to identify a hidden syndrome. BMC Pregnancy Childbirth.

[6] Williams A, Lavender T, Richmond DH, Tincello DG (2005). Women’s experiences after a third-degree obstetric anal sphincter tear: a qualitative study. Birth.

[7] Nilsson IEK, Akervall S, Molin M (2021). Symptoms of fecal incontinence two decades after no, one, or two obstetrical anal sphincter injuries. Am J Obstet Gynecol.

[8] Antonakou A (2018). The long-term physical, emotional and psychosexual outcomes related to anal incontinence after severe perineal trauma at childbirth. Eur J Midwifery.

[9] Brown S, Gartland D, Perlen S (2015). Consultation about urinary and faecal incontinence in the year after childbirth: a cohort study. BJOG.

[10] Department of Health and Social Care (2022). Women’s Health Strategy for England.

[11] Braun V, Clarke V (2022). Thematic analysis A practical guide.

[12] Hillman S, Bick D, Seers K (2023). GRACE — LearninG from Women’s ExpeRiences of Anal InContinencE after Vaginal Birth.

[13] Parsons J, Eccles A, Bick D (2023). Women’s experiences of anal incontinence following vaginal birth: a qualitative study of missed opportunities in routine care contacts. PLoS One.

[14] Ziebland S, McPherson A (2006). Making sense of qualitative data analysis: an introduction with illustrations from DIPEx (personal experiences of health and illness). Med Educ.

[15] RCGP Learning (2023). Anal incontinence in women with previous childbirth injury (eLearning module).

[16] Norton C (2004). Nurses, bowel continence, stigma, and taboos. J Wound Ostomy Continence Nurs.

[17] Rasmussen JL, Ringsberg KC (2010). Being involved in an everlasting fight: a life with postnatal faecal incontinence. A qualitative study. Scand J Caring Sci.

[18] Bugg GJ, Hosker GL, Kiff ES (2005). Routine symptom screening for postnatal urinary and anal incontinence in new mothers from a district. Int Urogynecol J Pelvic Floor Dysfunct.

[19] Brown HW, Wexner SD, Lukacz ES (2013). Factors associated with care seeking among women with accidental bowel leakage. Female Pelvic Med Reconstr Surg.

[20] Stormacq C, Van den Broucke S, Wosinski J (2019). Does health literacy mediate the relationship between socioeconomic status and health disparities? Integrative review. Health Promot Int.

[21] Yin HS, Dreyer BP, Vivar KL (2012). Perceived barriers to care and attitudes towards shared decision-making among low socioeconomic status parents: role of health literacy. Acad Pediatr.

[22] National Institute for Health and Care Excellence (2021). Postnatal care NG194.

[23] Lindquist A, Kurinczuk JJ, Redshaw M, Knight M (2015). Experiences, utilisation and outcomes of maternity care in England among women from different socio-economic groups: findings from the 2010 National Maternity Survey. BJOG.

[24] Smith HC, Saxena S, Petersen I (2020). Postnatal checks and primary care consultations in the year following childbirth: an observational cohort study of 309 573 women in the UK, 2006–2016. BMJ Open.

[25] D’Souza JC, Monga A, Tincello DG (2020). Maternal outcomes in subsequent delivery after previous obstetric anal sphincter injury (OASI): a multi-centre retrospective cohort study. Int Urogynecol J.

[26] Gurol-Urganci I, Cromwell DA, Edozien LC (2013). Third-and fourth-degree perineal tears among primiparous women in England between 2000 and 2012: time trends and risk factors. BJOG.

